# Integration of health care in the Basque Country during COVID-19: the importance of an integrated care management approach in times of emergency

**DOI:** 10.1017/S146342362100044X

**Published:** 2021-08-11

**Authors:** Julen Izagirre-Olaizola, Goizalde Hernando-Saratxaga, María-Soledad Aguirre-García

**Affiliations:** Department of Finance Economics II, University of the Basque Country (UPV/EHU), Leioa, Biscay, Spain

**Keywords:** Basque Country, COVID-19, health system, integrated care, public management

## Abstract

The main objective of this study is to analyse the process of integration of health care implemented in the public health system (Osakidetza) of the Autonomous Community of the Basque Country (CAPV), and assess whether the steps taken to date have helped or hindered the work of health personnel in times of COVID-19. Based on a case study, an assessment is made of the way in which certain tools of the integration process have been applied, if they have worked well and if they have led to better management of the pandemic.

For the purpose of this study, a qualitative methodology is chosen consisting of a case study and in-depth interviews with health personnel at the front line of the integration process and the fight against COVID-19.

This study makes two fundamental contributions. First, it analyses the health integration process in recent years in the public health system of the Basque Country. Second, it gathers the perceptions of different agents related to the Basque Health System of the way in which the tools of the integration process implemented in recent years have worked during the pandemic, detailing the positive and negative perceptions in this regard.

Our conclusions offer a series of strategic recommendations linked to comprehensive patient care and the use of tools related to teleconsulting: the unified medical record, electronic prescription, and non-face-to-face care channels.

## Introduction

The COVID-19 pandemic has infected millions of people and caused hundreds of thousands of deaths around the world. The World Health Organization (WHO) classified COVID-19 as a global pandemic in March 2020 (WHO, 2020). The health emergency has put the operation of health systems to the test and, in many cases, these have been overwhelmed by the number of hospital admissions, the lack of resources, or the need to take extreme measures of protection in the care of COVID-19 patients. But the pandemic has also led to problems when managing other patients with conditions unrelated to COVID-19 (Hong *et al.*, 2020; Lee & Morling, 2020).

Mortality from COVID-19 infection has been closely related to factors such as age or the pre-existence of health problems (Zhou *et al.*, 2020). In recent years, the ageing of the population and the demand for care due to dependency, disability, and chronicity are amongst the biggest challenges for public welfare policies and, ultimately, for the economic and social organisation of society (Singer *et al.*, 2011; Agnihothri *et al.*, 2020). These challenges, coupled with the health emergency caused by COVID-19, have put the health systems of various developed countries under enormous pressure.

Even prior to the pandemic, the increasing prevalence of chronic diseases and the complexity and cost of treatments highlighted the urgent need for a coordinated healthcare service (Ahgren & Axelsson, 2007; Valentijn *et al.*, 2013; Segato & Masella, 2017). This challenge is now greater than ever since the impact of the COVID-19 pandemic not only affects the care received by patients with the virus but has a knock-on effect on the general population and, in particular, chronic patients.

It is encouraging to see that the integration processes of the health system carried out in recent years have provided health personnel with some tools that have since become absolutely vital for managing the current healthcare demands. A particular area is teleconsulting which has been increasingly important in the health care of all types of patients (Agnihotri *et al.*, 2020; Ohannessian *et al.*, 2020; Perrin *et al.*, 2020; Wosik *et al.*, 2020). Teleconsultation, an exchange of medical information by electronic means, assesses symptoms in order to correctly direct the appropriate level of care and to minimise the number of consultations a patient needs. It centralises information in primary care, improves the quality of medical services, and encourages interrelation, collaboration, and coordination between different professionals and specialists. In addition, if the condition of the patient allows him/her to remain at home, information regarding the relevant care process is passed to the district nurse. Collaboration is the key with the overall aim of improving the quality of life of patients and reducing waiting time.

Now more than ever, public health services must recognise and accommodate patients’ individual medical needs, social settings, and care preferences (Singer *et al.*, 2011; Bettiga *et al.*, 2020; Wosik *et al.*, 2020). An orientation towards integrated care, which guarantees continuity of care and, in addition, places the person at the centre of the system (Algren & Axelsson, 2007) could provide better health management in critical health situations.

The paper begins with a description of the integration process of the Basque Health System. The main objective of this study is to describe the way in which the tools derived from the integration process have facilitated or hindered the performance of various agents of the system. To evaluate this, impressions are collected from various agents (medical and nursing staff, managers, patients, institutions …) and their perception about the advantages and limitations of the tools of the integration process in the context of the fight against the pandemic. The questions being asked are: how have the tools generated by the integration process been applied to the population affected by COVID-19? How do the different agents perceive the usefulness of this type of tool in caring for the general population in such a complicated context? Have these tools served to better manage the pandemic?

For this study, a qualitative methodology is applied consisting of a case study and in-depth interviews conducted with people who have worked with and, in some cases, initiated the integration process in recent years, and who have been on the front line in the fight against the COVID-19 pandemic.

This study makes two fundamental contributions. First, it analyses the health integration process carried out in recent years in the public health system of the Basque Country. Second, it gathers the perceptions of different agents related to the Basque Health System of the way in which the tools of the integration process implemented in recent years have worked during the pandemic, detailing the positive and negative perceptions in this regard.

## Context of the challenge: the health crisis caused by the COVID-19 pandemic

John Hopkins University (JHU) reported that deaths caused by COVID-19 exceeded 1 million on 29 September 2020, only 10 months after the first confirmed death in China. By March 2021, this figure was already approaching 3 million (JHU, 2021).

Since then, the effect of the pandemic has gone beyond a health crisis to become a global social and humanitarian crisis. From a health management point of view, huge organisational disruption has been seen across various health systems (Ohannessian *et al.*, 2020). This disruption has occurred at different levels, from direct care of those affected by the SARS-CoV-2 virus to the day-to-day health care of the general population, and has been exacerbated by social distancing and the preventive measures adopted.

The challenge has been how to meet, under extremely complicated conditions, the urgent and immediate demands caused by the pandemic but without neglecting population health care. In many cases, the effect of COVID-19 meant other health care has been reduced to a minimum, leading to cancelled appointments, postponed operations, and even patients with relatively important and urgent pathologies not being treated or not maintaining their care plan (Byrne, 2020; Smith *et al.*, 2020; Zintsmaster & Myers, 2020).

Obviously, this disruption in health care leads to significant health and social problems. Once the initial lockdown and social distancing measures had been relaxed slightly, health systems faced the challenge of resuming normality for patients affected by pathologies other than COVID-19.

In this context, the use of health management tools that minimise face-to-face contact has increased enormously.

However, the sudden wider adoption of these tools, which were already available, needs to be combined with the development of comprehensive health management strategies that use these and other tools to offer better health care in an economically sustainable way. For this reason, we want to analyse the potential of integrated health care as a strategy for meeting the structural challenges going forward and bringing about the transformation required, even in dramatic situations such as a pandemic.

## Structural challenge: integrated health care

### Definition of integrated health care

The current critical situation should not serve as an excuse to compromise quality and efficiency in the care of the general population. More than ever, patient autonomy is central to this. The concept of healthcare integration must put the user of health care at the centre of the system. Integrated patient care can be defined as ‘patient care that is coordinated across professionals, facilities, and support systems; continuous over time and between visits; tailored to the patients’ needs and preferences; and based on shared responsibility between patient and caregivers for optimising health’ (Singer *et al.*, 2011).

This definition emphasises the need to acknowledge the patient’s preferences and capacities for self-care, rather than simply meeting his/her medical needs. Other definitions of the patient-centred approach emphasise access, dignity, and respect, information exchange, participation, simplification, and coordination as key objectives (Davis *et al.*, 2005). From a healthcare integration point of view, tailoring care to improve patients’ health requires caregivers to shift from seeing patients as passive recipients to active participants in their care (Rittenhouse & Shortell, 2009). In this sense, the concept of ‘self-management’ has been widely used in the field of health management, especially related to the training and education of patients with chronic diseases (Lorig & Holman, 2020). For these types of patients, the management of their disease becomes a lifelong task and they need to approach it from a stance of maturity, education, and responsibility, but supported by a system that facilitates such management.

### The need for a revised model

In recent years, the need for a profound process of adaptation of the health system in many advanced societies (as in the case of the Basque Country) has become increasingly evident (Jauregui *et al.*, 2016; Hernando-Saratxaga *et al*., 2021). The constantly ageing population and the rise in the number of patients with chronic and multi-pathological conditions have meant a review of the system is essential. The pre-existing model, originally conceived more for the care of acute patients, does not meet current structural needs (it can adapt well to acute patients due to COVID-19 but neglects the correct functioning of the system in general).

A reorientation of the structural model needs to consider three fundamental factors: (i) the demographic context of the Basque Country (and other similar contexts), whose population has remained stable in recent years, but with one of the highest life expectancy levels in the world and the consequent tendency to ageing; (ii) the increase in chronicity, both due to the ageing of the population and because many diseases have become chronic; and (iii) the economic context, where health costs have risen notably over the last decade (BHD, 2017). Clearly, these three structural issues are compounded by the current, although possibly cyclical, challenge of the COVID-19 pandemic.

### Organisational change and development of integrated care in the Basque Country

#### The structural process of healthcare integration in the Basque Country

To structure the analysis of the results of the integration process, the model proposed by Wagner *et al.* (1998), *The Chronic Care Model* (CCM) is used which is the conceptual framework employed by the Basque chronicity strategy (BHD, 2010) since 2010. In this model, chronic care is addressed in three areas: the community, the health system, and the patient. The main key elements in their application in the Osakidetza integration process are discussed below.

The new approach supposes a profound transformation of the care and management model, which is too fragmented and based on care delivered in disjointed appointments that do not offer the continuity and the transversality of care necessary for good management of chronic patients. A patient-centred model is proposed that can provide continuity of health and social care, facilitating new structures, processes, and tools that allow health needs to be met in an effective, more efficient, and more coordinated way by health and social professionals. This has the potential of preventing unnecessary hospitalisations and, as a consequence, reducing economic, personal, social, and opportunity costs (BHD, 2017).

The change described above took place in the Basque Country through the creation of a new organisational structure called Integrated Health Organization (IHO), which aims to promote multidisciplinary, coordinated, and integrated care between the different services and levels of care, particularly promoting collaboration and organisational integration between primary care and specialised care (Toro Polanco *et al.*, 2015). The healthcare structure went from a general structure made up of 35 organisations independent of each other (15 hospitals and 20 primary care health districts with an additional 475 outpatient health centres), in 2010 to one made up of 18 organisations that include care centres, and inpatient and outpatient hospital care (13 OSIs, 2 hospitals, and mental health networks). They share management, objectives, strategies, and information about all the people they treat. Financial economic management is also shared. The aim is to achieve intermediate results such as better patient control, coordination, and continuity of care and interprofessional collaboration, and end results related to improved patient satisfaction and greater efficiency (Vázquez *et al.*, 2012).

Given the objectives of this study, we focus the analysis on those tools used in the health integration process and which allow better health care in times of a global pandemic.

#### Integrated functional care framework

As mentioned earlier, the new Basque Health Model takes the form of 13 new organisational structures called IHOs, in addition to 2 hospitals and 3 mental health networks.

IHO are networks of health services that offer coordinated care through a continuum of benefits to a specific population and that are responsible for the costs and health outcomes of the population. They are set up around a regional hospital, and are responsible for the coverage of a range of services of primary care, specialised in social health care, for a geographically defined population, which is allocated in their IHO network.

The objective of any IHO or service network is the overall efficiency of the provision and continuity of care, through an intermediate objective: the improvement of the coordination of services to ensure that there is crossover in the care a patient receives from multiple sources of provision. IHOs are generated through vertical (primary and specialised care) and horizontal (care at the same level) integrations. In addition, they directly provide all the services they offer and have the same management and objectives for all the providers that make up the network. Each IHO is assigned individual financing in order to encourage all providers involved to work together to ensure all requirements are met within budget, and that treatment of the health problem is directed to the most appropriate department within the continuum of care to reduce costs and strengthen the quality of services (Vázquez *et al.*, 2005; 2012).

IHOs prioritise treating chronic patients outside the hospital. This makes savings possible such as a reduction in hospitalisations and re-hospitalisations, on spending on drugs, and on the number of visits to the emergency clinic. This model requires the gradual ‘shifting’ of resources towards the home/community setting and towards primary care.

The search for improvement in continuous patient care requires coordination between areas that historically worked in parallel but with little interaction between hospital and primary care area, so that they harmonise and achieve a common goal without conflict. When the coordination of the continuity of care reaches its maximum degree, care is integrated. Thus, continuity of care, defined as the degree of coherence and union of experiences in care perceived by the patient over time, requires continuity and coordination in the management, availability, and use of information in the relationship and interaction of the patient with the care provider over time (Vázquez *et al.*, 2005). To do this, and to break with the traditional compartmentalisation, mechanisms and tools have been used that favour this coordination. These are described below.

#### Integrated Information and ICT Systems

##### Unified medical records and shared pharmaceutical history

Thanks to the new unified medical record, health professionals can access the patient’s medical history in primary care, hospitals, and out-of-hospital outpatient clinics. This improves care coordination and ensures information is fully updated and shared, leading to better therapeutic decisions for the patient.

The generalised assessment of the unified medical record and shared pharmaceutical history is very positive, and is acknowledged by health professionals as they are playing a central role in the process of integration and person-centred care. The exchange of data between all professionals aids greater security, fosters communication, and leads to faster diagnosis and greater clinical precision.

##### Non-face-to-face consultation

Non-face-to-face consultation between professionals is another tool with great potential. These consultations improve activity in primary care by providing the patient with a faster response and avoiding unnecessary trips to hospital centres, with the professionals from two or more levels working in a coordinated manner.

##### Osarean (non-contact channels)

Osarean is a multichannel health services centre whose mission is to promote accessibility and improve service to citizens by offering a hub through which patients, families, and professionals can interact with Basque public health in a remote way: by phone, email, videoconference, TV, web, social networks, etc.

These new channels facilitate active patient self-management and help meet the needs, not previously covered, of patients with chronic diseases, providing tools to assist professionals in offering improved care.

#### Collaboration in community and socio-health spheres

Strong links with the social health sphere are seen as fundamental in a context in which social and care needs are increasing.

Joint work with associations, city councils, sports centres, etc., helps reinforce prevention and education, taking health care out of the exclusive healthcare field, and improving the training and health pedagogy of citizens so that they can adopt a more active and positive role in their own health care.

#### New role of citizens and the active patient

The guidance that health personnel offer to their patients leads to more active patients who are better prepared to manage their disease. They feel more empowered with greater autonomy in deciding how to improve their own health. There is consensus amongst the people interviewed, professionals and patients, about the need to work on the development of more active patients. We highlight some statements made by several of the healthcare professionals interviewed:

*“we must recover the maturity of the patient. He/she must be given a central role. We healthcare professionals should talk to the patients, explain things, ask their opinions and even make decisions with them about what they want or do not want us to do and how far they want to go in any treatments and interventions”.*

## Study method and design

The methodology we use in this study is qualitative and consists of an in-depth study of a case related to the integration of the Osakidetza-Basque Health System. To this end, interviews are conducted with people who have worked with and, in some cases, initiated the integration process in recent years, and who have been on the front line in the fight against the COVID-19 pandemic.

People with positions of responsibility and management in Osakidetza are interviewed by the research team on two occasions; before, and after, the onset of the pandemic. In this phase of the study, a total of 20 people with different profiles are interviewed.

The existence of an ongoing project was used to study the integration process, to look more closely at the implications of the process for health personal after the first few months of the pandemic. Therefore, the interviews with the same personnel were repeated and expanded, although some people only participated in the first series, and others only in the second. An in-depth study and analysis are carried out based on two axes; the integration process (first series of interviews) and its impact at the time of a health crisis due to COVID-19 (second series of interviews).

The objective was to interview people who came from very different fields of work; medical, nursing, management, primary care, hospital care, care for patients and caregivers, etc. Table 1.

**Table 1.** Classification of the people interviewed

Specific person interviewed	Management	Primary care	Hospital care	Caregivers/patients
Manager of Coordination of medium-sized IHO	X			
Head of Ministry of Health	X			
Head of Territorial Mental Health	X			
Manager of Innovation in Management	X			
Manager of Innovation in Management	X			
Primary doctor and Head of Primary Health Care Unit	X	X		
Hospital Pulmonology Specialist			X	
Internist			X	
Pulmonologist/Head of the Hospital Pneumology Service	X		X	
Physician and Head of Emergency Service, medium/long-stay hospital	X		X	
Internist/Head of Hospital Internal Medicine Service	X		X	
Hospital Liaison Nurse			X	
Advanced Practice Nurse		X		
Nursing Assistant		X		
Retiree, Patient Caregiver, Neighbourhood Association				X
President of Basque Retirees Association				X
Head of the Diabetes Association				X
Head of the Primary Care Unit and Member of the Osatzen Board, Basque Society of Family Medicine	X	X		
Head of the Hospital Pulmonology Service	X		X	
Primary Care Doctor		X		

Source: Compiled by the authors.

### A systematic method of analysis:Tthematic Analysis

The qualitative study is based on 20 open, in-depth, and semi-structured interviews with the aim of exploring the subject matter. The analysis is focused on speech and non-verbal behaviour. The research group is made up of three people. Two members of the research group attend every interview. The two researchers follow a script in the interview, which is held at a determined meeting place, and the conversation is recorded for later analysis of speech and non-verbal behaviour.

The interview script is prepared from the in-depth interviews and meetings that the research team holds with Osakidetza managers.

After conducting the interviews, a wealth of qualitative information is generated. Qualitative methodologies enable clarification of a complex phenomenon through a process of exploration. The use of semi-structured interviews allows the maximum amount of information to be gathered in a systematic way, but without losing the wealth of opinions and experiences (Belotto, 2018).

In order to minimise the risks of an excessively vague or subjective analysis, we follow a systematic method that allows the analysis to be carried out in depth but with all the methodological guarantees. Thematic Analysis is ideal because it allows the analysis to be carried out with accessibility and flexibility. Thematic Analysis is a method for systematically identifying, organising, and offering insight into patterns of meaning (themes) across a data set. It allows the researcher to see and make sense of collective or shared meanings and experiences (Braun & Clarke, 2012).

The validity of this tool for the content analysis of in-depth interviews such as those carried out in this study has been endorsed by various authors (Braun & Clarke, 2006; Thomas & Harden, 2008; Vargas-Halabi *et al.*, 2015; Belotto, 2018; Lawless & Chen, 2019).

Although it is not possible to show in detail the entire process carried out in a study of these characteristics, it is important to follow a series of steps that guarantee the quality and rigour of the results presented (Belotto, 2018; Thomas & Harden 2008).

There are different approaches regarding the specific steps to be followed, although, in this study, we have applied the steps suggested by Braun and Clarke (2006; 2012).

Table 2 shows the six steps or phases proposed by these authors, and the approach taken by the researchers in carrying out this analysis.

**Table 2.** Phases of the Thematic Analysis and steps followed in the analysis of the information

Phase 1: Becoming familiar with the data	The first phase consists of transcribing, without delay, the verbatim interventions of the participants, the researcher’s interventions, and a set of notes in which an attempt is made to reflect various aspects. The job of transcribing was shared between the researchers, with each researcher being responsible for transcribing the interviews in which he/she was not present. All the investigators read, and reread, all the transcripts.
Phase 2: Generating initial codes	The subject matter was established and the information from all the fragments of each interview was categorised in codes or blocks of interest, extracting basic concepts that emerged. Initially, each researcher tries to extract their own codes. Although analysis software could be used, we prefer to carry out this process manually which, although there is a lower level of automation, makes for a more personalised and richer interpretation (Braun & Clarke, 2012; Belotto, 2018)
Phase 3: Identifying ideas	In this phase, the aim is to find common ideas, combine codes and, at the same time, verify that the perceptions of the different researchers are coherent, reducing dissonances and reinforcing the results. The categories and codes are provisional, and are constantly being reviewed (eliminating some, merging others, proposing new ones …) until completion of the analysis.
Phase 4: Reviewing themes	In this phase, we try to refine the ideas obtained in the previous phase and, in turn, attempt to map out conceptually the most important latent and recurring themes that would be consistent with the approach of the interviews and the data set obtained.
Phase 5: Defining and naming themes	This phase focuses on giving meaning to each theme, establishing logical connections between the defined categories, and trying to reduce interferences between them. This is a complex step since, in many cases, the contents are closely linked, making it difficult to design a final map that is easy to understand but, at the same time, faithful to the data obtained (see Figure 1).
Phase 6: Producing the report	Once all the previous steps have been carried out, the next step is to describe the scenario behind each theme in a faithful way, reinforcing and enriching it with representative textual quotes extracted from the interviews. The result of this last phase is presented in section 5 of this paper.

Source: Compiled by the authors using the steps proposed by Braun and Clarke, 2006; 2012.

## Results

### Presentation of the conceptual map of the results obtained

As indicated in the methods section, the content of the interviews carried out is analysed through the use of the qualitative analytical method called Thematic Analysis. Thematic Analysis is a method for identifying, analysing, and reporting patterns (themes) within the data. It organises and describes the data set in (rich) detail (Braun & Clarke, 2006).

**Figure 1.**
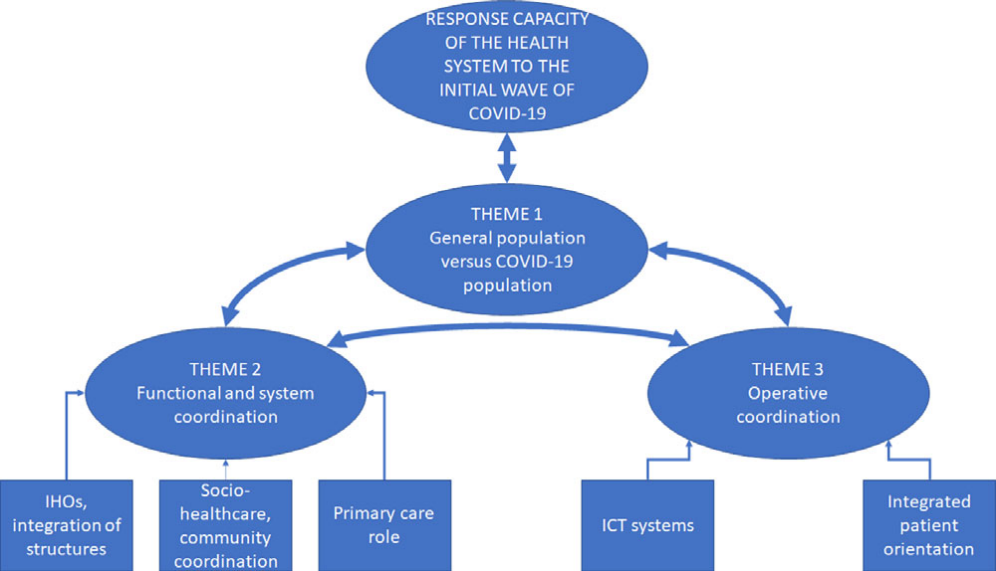
General concept map of the study: three main themes of analysis. Source: Compiled by the authors.

After completing a systematic and disaggregated analysis of the information, through the previous phases defined in the methodological section – (1) reconstruction of the phenomenon, separating what is important from the superfluous; (2) initial code generation; (3) identification of basic ideas; (4) review of themes; and (5) definition and naming of themes and relationship of codes – a thematic map is developed from the information obtained from the in-depth interviews (Figure 1).

In short, a preliminary work is completed on the identification and systematic classification of ideas and concepts in line with the required quality criteria for analysis (Belotto, 2018). Although it was difficult to isolate three main dimensions due to the constant interrelationships that emerged, the steps followed enabled the results to be presented in a valid way and with data that supported this concept map.

### Theme 1: response offered to the general population versus the population directly affected by COVID-19

**Figure 2.**
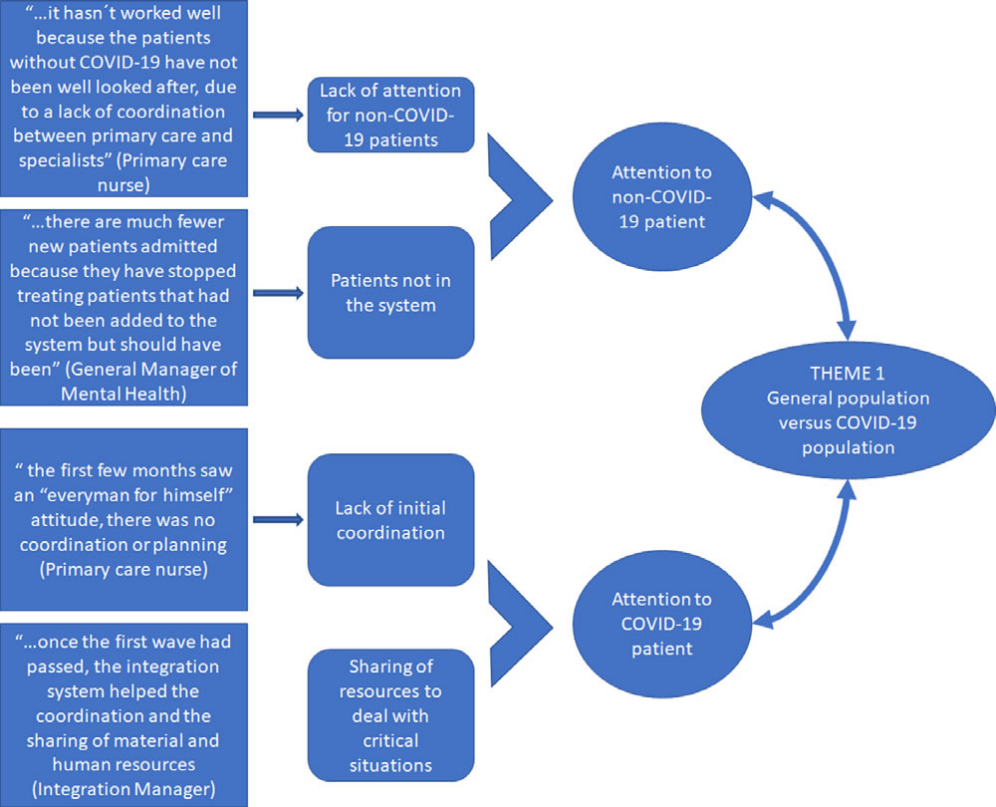
Analysis of Theme 1: health care of the general population and COVID-19. Source: Compiled by the authors.

A recurring theme in all the interviews analysed is the treatment received by patients in times of a pandemic. A clear distinction reported in all interviews was the separation between patients affected by COVID-19 and health care for the general population during the pandemic (Figure 2).

In the analytical dimension related to the COVID-19 patient, opinions are quite similar in all cases. An initial key and recurring idea are the sense of urgency and overflow of pre-existing structures. The first wave of the pandemic completely exceeded the capacity of the Basque Health System (like so many others around the world), which pushed healthcare personnel of all kinds to extreme situations, both from a work and personal perspective. The lack of foresight is also emphasised, and the need for greater investment in health and, specifically, in epidemiology. Once the first wave had passed, health personnel were in a better position to review the IHO system, and begin to assess how the integrated health system had functioned as a response tool to the pandemic. The general view is that formal integration into IHOs facilitated the transfer of resources, both human and material, which made a more flexible response to the pandemic possible. However, there are also those who consider that integration has not translated into the coordination desired and that, in many cases, response mechanisms were not sufficiently agile.

The health personnel in their interviews also place great importance on the health care of the **general population**, those who are not sick with COVID-19 but obviously still need a variety of healthcare services. The most frequent opinions refer to the lack of attention that the general population has received, either because the health system could not absorb the workload, or because of the restrictions that led to minimal care and in worse conditions (reducing presence to a minimum), or even because in many cases the patient himself chose not to go to the health centre, given the health emergency situation. This scenario could lead to long-term problems, and most professionals advocate seeking, as far as possible, an appropriate balance between telecare and a more personal approach, face-to-face, ensuring a more inclusive treatment.

### Theme 2: structural–organisational integration and its operation during the pandemic

**Figure 3.**
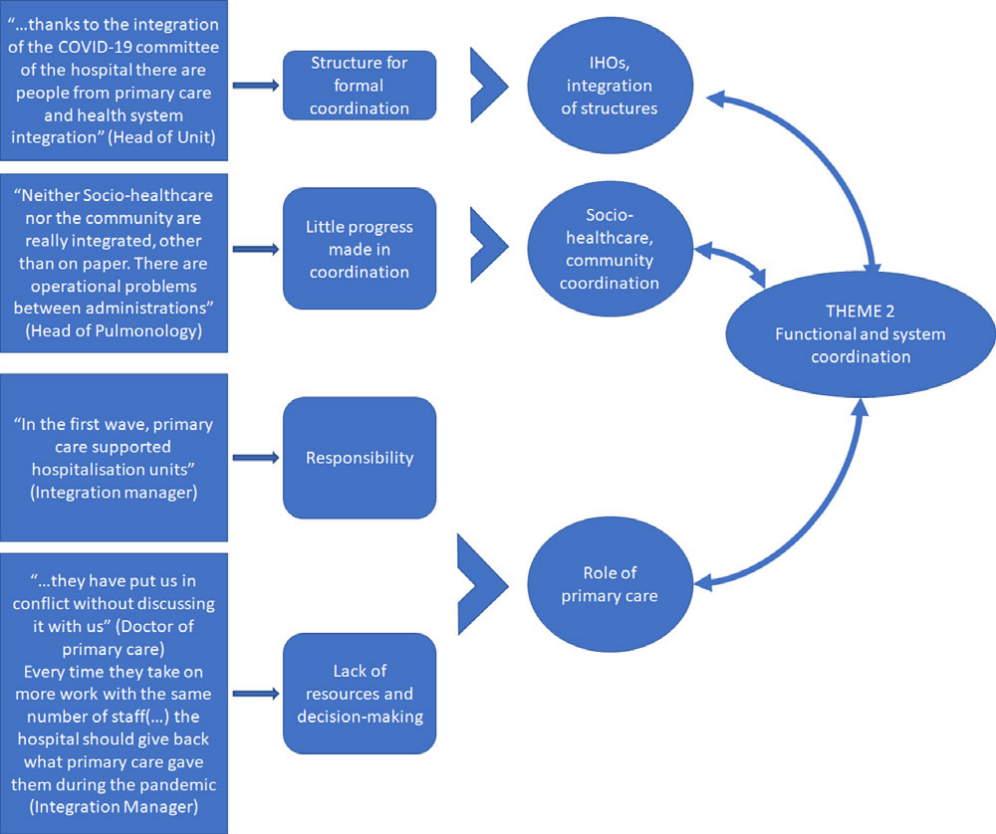
Analysis of Theme 2: structural and system coordination. Source: Compiled by the authors.

The second major theme that brings together many quotes-concepts-ideas in the data collected is related to the process of structural integration of the Basque Health System, and the way it has influenced the response to the COVID-19 pandemic. Following the systematic analysis of the information, three dimensions can be distinguished within this block: the actual integration of structures, the transformation of the role of primary care in the health system, and the relationship of the health system with the community and socio-healthcare (Figure 3).

Interestingly, **structural integration** is the aspect that has generated the most feedback. Several professionals claim that the integration of structures has facilitated coordination and the transfer of resources from underused areas to the front line of the fight against the pandemic:

*“a great advantage of being an integrated organisation is that we have been able to relocate part of our staff to spaces where they were needed”. “The available resources have been better used; being integrated, it has been easier to share resources”.*

In addition, the integration helped in that ‘*working as one organisation made it easier to create a COVID-19 crisis committee and information was shared more easily’.* ‘*Without integration, we would have had less communication and things would have been worse’*. In the past, this coordination and this transfer would have been impossible or, at least, much more complicated.

The opinions of the professionals consulted are, in general, positive about the new organisation in IHOs and other structures. In the main, IHOs are thought to facilitate integration between levels of care, promoting care in the most appropriate place that best adapts to the needs of patients. This is the view of one primary care physician: ‘*They are necessary. We had two different realities*, with different objectives, different directors … yet we were working for the same patient’. And a manager from the Basque Government’s Health Department stated that ‘*the change of organisational structure is not an objective in itself, it is intended to be simply an element that facilitates integration*’.

The other major themes that featured in practically all of the interviews carried out is **the role that primary care should play** in this process of structural integration, how this has been articulated in times of pandemic, and how the future should evolve. Both primary and hospital care recognise that the fight against COVID-19 has been carried out, above all, by primary care (except, obviously, the most serious cases that required hospitalisation and resulted in hospital emergency units being overwhelmed). At this point, although there is unanimous consensus that this debate should be resolved, the perceptions are very different depending on whether the people interviewed belonged to primary or hospital care.

Those professionals working in hospital care recognise that primary care is assuming a large part of the fight against COVID-19 and that it should play a fundamental role in health care in general, which is vital for a good integration process. In addition, several voices emphasise that integration in IHOs allows the presence of primary care in decision-making and coordination bodies. In the words of a hospital internist: ‘*There is an understanding between professionals and that makes things easier on a day-to-day basis*.’ Furthermore, in the words of a manager in charge of pulmonology in a hospital, ‘*this improves communication and ensures the patient is not lost in the system*’.

However, the opinions received from primary care medical and nursing personnel have been very harsh and very critical in this regard. On more than one occasion, it is denounced that integration into IHOs has meant a total concentration of power (and with it decision-making capacity and budget allocation) in the hands of hospital care. This implies, according to primary care personnel, that primary care under the control of the IHOs assumes more and more tasks without the decision-making capacity or adequate human or material resources. This is where the strongest criticism is heard from people who consider that the integration of structures has meant a great imbalance, that decision-making and the allocation of resources have been left to the hospitals, with primary care seeing a loss in resources and decision-making capacity over recent years. In the words of several of the people interviewed,

*“Integration will drown out the voice of primary care because, when the main manager is the hospital, the money and resources go to the hospital. The hospital should work at the pace established by primary care”.*

Thus, a health professional concludes that

*“the health system has to offer strong primary care: the pyramid of healthcare should have its foundation based in primary care but, with the creation of IHOs, the pyramid is being inverted”.*

Lastly, there are also many doubts regarding **coordination with the socio-healthcare and community sphere**. Although there is consensus that this coordination should be improved, all those who speak on this issue believe that there has really been little progress in this direction. In fact, this lack of coordination is considered to have hampered a better response to the pandemic. Some administrations did not react fast enough which led to complicated situations, for example, in nursing homes, forcing the healthcare system to take over tasks, further overloading the system. In the words of the Osakidetza integration director:

*“Osakidetza reacted quickly and took on tasks that should have belonged to the Provincial Council. An overload. They took on tasks related to the additional patients as well as nursing home staff. The management of the private companies left a lot to be desired”.*

Over the course of this study, various concerns were voiced, such as:

*“We have a terrible problem and it is going to get worse. People are living much longer now, but are much more dependent. Discharging many 80-year-old patients from hospital is extremely problematic. If they are in a residential home it is easy, but if they live alone it becomes a serious social problem rather than a medical one”.*

It is clear that a number of those interviewed agree that much remains to be done to guarantee the best social and health care for citizens. There is a proliferation of institutions and stages that slow down many necessary procedures and processes.

*“It is important that the social background of the patient is included in the unified medical record so that health workers know things about the patient’s life, where they live, their family, who helps them etc.”*

### Theme 3: operational coordination, concrete tools for the common fight against the pandemic

**Figure 4.**
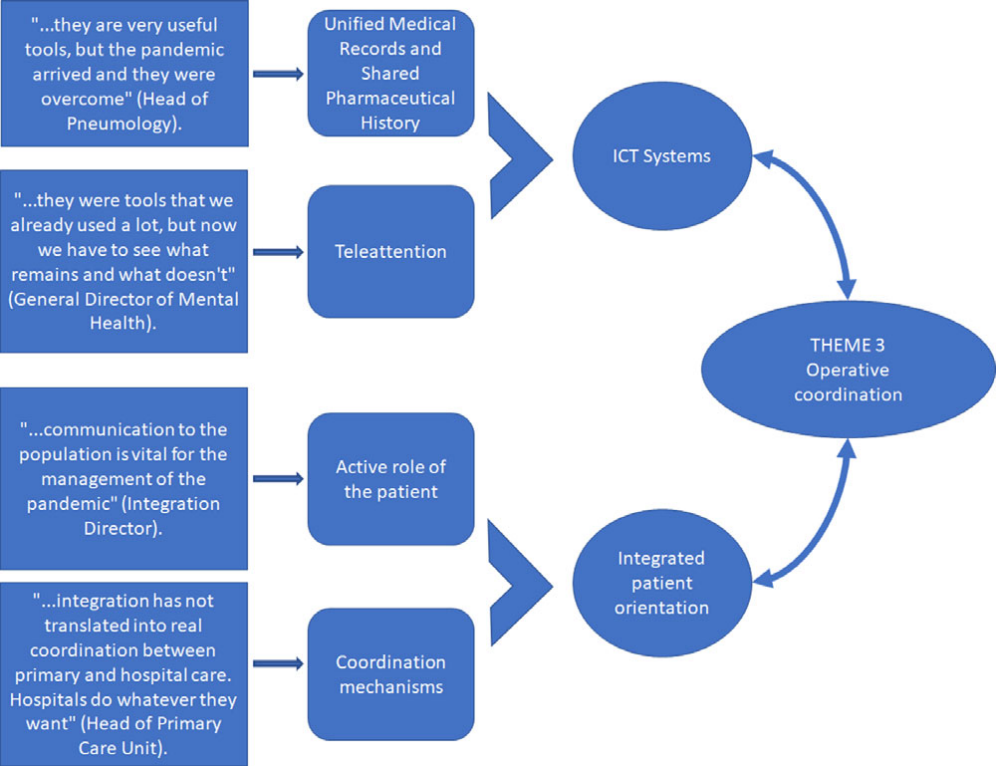
Analysis of Theme 3: organisational coordination, centrality of the patient. Source: Compiled by the authors.

Finally, the third thematic block identified in the systematic analysis of the interviews is that related to organisational coordination, that is, the use of specific coordination tools between services that are in support of putting the system at the service of the patient, and not the other way round (Figure 4).

Opinions regarding coordination are divided into those related to ICT tools that allow better coordination, and the centrality of the patient through an active role and the coordination between specialists.

In general, the professionals consulted point to the importance of having ICT tools that can facilitate the health management of the population in a context of social distancing. Tools such as telephone assistance, remote consultation, or electronic prescription are valued very highly. The use of these tools has become increasingly important in a crisis situation and it seems they will be here to stay even after the end of the pandemic. The professionals affirm that,

*“it has been possible to attend to all patients under teleconsulting and telephone consultation modalities. Theses are tools that we used before but whose use has been greatly enhanced by COVID”. “Without the electronic prescription, everything would have been a disaster during the pandemic. It has been a vitally important tool”.*

Although there is a unanimous consensus that face-to-face care cannot be substituted, there are certain procedures that could continue to be carried out remotely in the future, without reducing the quality of care, and that would help to alleviate the waiting time for certain services. However, it is also believed that the health emergency has highlighted inefficiencies in the system, the lack of development of some tools, and a serious lack of resources to deal with situations of this type, mainly because current programmes were created to deal with a traditional approach and not to deal with a global crisis,

*“and, then, what happens is that the pandemic has gotten them out of control. We have had to adapt quickly to offer attention at a distance and this has brought problems. The ideal would have been to prepare these tools better”.*

In regard to unified medical records and shared pharmaceutical history, a nurse case manager said,

*“Being able to share everything that is done at all stages and by professionals with the patient is very useful (…). It´s important for everyone, but in primary care it has been particularly essential. It allows all professionals to have a common focus and share patient information (…). It has facilitated communication between different professionals who do not work together. This benefits the patient and ensures their process is better followed and better controlled”.*

In addition to these important advantages, health professionals also recognise that

*“many mistakes have been avoided; and it provides transparency, security, control and cost control. We all see what the patient is taking and if they are really taking it or not and if they are taking it properly, in the right doses, at the right time, etc.”*

In times of a pandemic, professionals agree on the importance of having previously worked towards the **empowerment of patients** and their active participation in managing their needs. This has shown that, regardless of the reorganisation carried out, a shared responsibility between the general population with a more empowered and responsible ‘attitude’ and their health workers and caregivers has been crucial in limiting the number of admissions.

However, in this regard, according to some of the people interviewed, there is still a long way to go. They agree that it is vitally important to continue working to achieve a more active patient and more awareness of what this context entails, where

*“the true magnitude of the problem must be explained to patients. They aren’t witness to the death, pain and exhaustion seen by professionals and the stress they are under. Communicating this to the population and involving them is vital”.*

Regarding new channels of coordination between professionals, such as non-face-to-face consultations, in general, the opinion of the professionals interviewed is positive, evaluating favourably the potential of the tool, but its application is perceived as having room for improvement. Several of the people interviewed state that,

*“The tool is interesting and valuable and is very instructive. It is used for non-urgent enquiries and cuts down on unnecessary travel. It also has the advantage of speed, but the disadvantage of a lack of traceability and more so when many professionals are involved. It is not very clear who said what or prescribed what, and since we are all a bit concerned with patient complaints and reports and sometimes we practise “defensive medicine”, the more that is written down, the better”.*

There are, however, also critical opinions regarding coordination in times of a pandemic:

*“it is a very good tool, but it was suspended during the pandemic, then it was resumed with difficulties, and right now it represents another delay for specialised care rather than a coordination tool”.*

## Discussion

The process of integrating health management is essential for quality and sustainable health care at any time, but especially in times of a health emergency, such as COVID-19. It is more important than ever to move towards a model that places patients at the centre of the system and, at the same time, educates and shares responsibility with them. The integration of self-management in health care must address, according to the people interviewed, challenges related to the transformation of the system, communication, and patient education, and the provision of necessary resources. These perceptions are consistent with previous studies that identify these key needs (Barlow *et al.*, 2002; Lorig & Holman, 2020).

The current emergency has meant that teleconsulting is being taken more seriously and has accelerated what had been a very slow implementation (Gyorffy *et al*., 2020; Smith *et al.*, 2020; Vilender *et al.*, 2020). Political and health authorities have been forced to work with this, and even patients have been more than willing to accept teleconsulting services (Hong *et al.*, 2020). Technological advances in recent years have made a wide range of digital tools available for use.

Despite its many benefits, for telehealth to survive as a viable alternative and not just in an emergency situation, the barriers that prevent its expansion and maintenance need to be addressed and structural changes must be implemented from the perspective of health systems management (Byrne, 2020; Ohannessian *et al.*, 2020; Smith *et al.*, 2020).

Although the integration process itself has been well accepted by the people interviewed, the appearance of the COVID-19 pandemic has highlighted serious inefficiencies and gaps that must be addressed to ensure the work towards a responsible patient, informed and with decision-making capacity, does not become lost in a system that collapses under emergency situations. Analysis, in section 5, of the data obtained through the interviews in this study has revealed serious doubts, especially on the part of primary care personnel, about the way in which the integration of structures has been carried out. There is consensus on the need for strong primary care that supports the integrated care of the patient, but doubts and complaints arise regarding the budget allocation or the lack of decision-making capacity and influence of primary care itself.

A formal integration process is nothing more than a vehicle, a tool for achieving the ultimate goal: coordination. The main critical opinions, especially from primary care, are related to the need for true coordination between services. The following opinion from a primary care physician reflects this concern well:

*“it is not worth mixing coordination with integration (…). The hospital should work for primary care (…) Here they come and tell you: the dermatologists have said that this has to be done like this. But let’s see, we´ll talk to the dermatologists and together we can decide what is best for the patient, right? Despite this integration process, they continue to impose on us how things should be (…) Hospitals do whatever they want. Despite the theoretical plan, we continue the same as always, because nothing is put into practice”.*

In times like these, primary care is the front line in health care and in the fight against COVID-19. For this reason, any integration process that aspires to serve as a valid framework for sustainable and quality health care must establish bridges and achieve a balance between the different levels of care.

## Conclusions: the need for real coordination

The global health emergency brought on by the COVID-19 pandemic has highlighted a series of shortcomings and inefficiencies in the health system. Although this pandemic occurred suddenly with widespread effects and is likely of a temporary nature, the idea that this type of health alert may be something cyclical and, therefore, an ongoing structural threat to health systems should not be ruled out.

Many of the aspects analysed have facilitated population health care in the worst moments of the health crisis, taking a leading role that is likely to continue (e.g., the rise of teleconsultations.

The whole process of integration and the shift towards a system that seeks to combine quality care and economic sustainability involves providing the health professionals with the resources and, above all, tools aimed at effective coordination. This need is especially relevant in the case of primary care, which is the first service to come under pressure both from the conjunctural (the emergency caused by COVID-19) and structural point of view (health care for the population, reducing hospitalisations, spending on medicines, and abuse of emergencies).

The integrated care system of the Autonomous Community of the Basque Country is making progress in meeting the current and future challenges of health care for citizens, but there is still a long way to go. The system presents some shortcomings and imbalances that must be tackled, especially in a context of increasing dependency, chronicity, and ageing, not only of patients but also of professionals, which exacerbates any health emergency.

A global concept of health must be adopted and this needs a coordinated and transversal effort from the health system and other administrations and social agents. This means a focus on a more active role for people in the care of their health and the management of their disease, where appropriate, and them taking on co-responsibility for the way they use health services.

To meet this challenge, the overriding objective of the health system must be to achieve real and effective coordination between different levels of care. Structural integration makes it easy to harness the potential of many healthcare integration tools but, fundamentally, it should serve as a catalyst for effective coordination. Primary care must be provided with sufficient resources, adequate conditions, and above all, decision-making and management capacity to be able to deal with extreme situations. If the health integration process seeks to promote health by trying to reduce hospitalisations, it is essential to have a strong, organised primary care with a voice that reflects its responsibility and the demands placed upon it.
